# Prognostic role of C-reactive protein to albumin ratio in cancer patients treated with immune checkpoint inhibitors: a meta-analysis

**DOI:** 10.3389/fonc.2023.1148786

**Published:** 2023-05-05

**Authors:** Menglu Dai, Wei Wu

**Affiliations:** Clinical Laboratory, Huzhou Central Hospital, Affiliated Central Hospital of Huzhou University, Huzhou, Zhejiang, China

**Keywords:** CAR, immune checkpoint inhibitors, meta-analysis, prognosis, clinical management

## Abstract

**Background:**

There are numerous articles investigating whether C-reactive protein to albumin ratio (CAR) is significant for predicting prognosis of cancer cases receiving immune checkpoint inhibitors (ICIs), whereas the results were inconsistent. We thus retrieved the literature and conducted the present meta-analysis for clarifying relation of CAR with survival outcomes among ICI-treated cancer patients.

**Methods:**

Through search against the Web of Science, PubMed, Cochrane Library, and Embase databases was carried out. The search was updated on 11 December 2022. This work later determined the combined hazard ratios (HRs) together with 95% confidence intervals (CIs) for estimating CAR for its prognostic efficiency for overall survival (OS) and progression-free survival (PFS) in cancer patients receiving ICIs.

**Results:**

A total of 11 studies consisting of 1,321 cases were enrolled into the present meta-analysis. As revealed by combined data, the increased CAR level markedly predicted dismal OS (HR = 2.79, 95% CI = 1.66–4.67, *p* < 0.001) together with shortened PFS (HR = 1.95, 95% CI = 1.25–3.03, *p* = 0.003) among carcinoma cases using ICIs. The prognostic effect of CAR was not influenced by clinical stage or study center. Our result reliability was suggested by sensitivity analysis and publication bias test.

**Conclusions:**

High CAR expression showed marked relation to worse survival outcomes among ICI-treated cancer cases. CAR is easily available and cost effective, which can be the potential biomarker for selecting cancer cases benefiting from ICIs.

## Introduction

Cancer refers to one of the most lethal diseases with high morbidity, mortality, and economic burden around the world (1). In recent years, immunotherapy has played pivotal roles in cancer treatment (2). Immune checkpoint inhibitors (ICIs) including antibody drugs that target programmed death-1 (PD-1) and programmed death ligand-1 (PD-L1) can provide a durable clinical response among diverse cancers in the case of effective treatment (3). ICIs such as nivolumab (anti-PD-1), atezolizumab (anti-PD-L1), or ipilimumab [anti-cytotoxic T lymphocyte–associated antigen 4 (CTLA-4)] have shown prolonged survival against cancer (4–6). It is important to note, however, that only a small proportion of patients can benefit from ICIs, and others do not respond to immunotherapy, thus limiting their use in the clinic (7). Consequently, identifying effective prognostic biomarkers for predicting the survival of cancer patients undergoing ICIs is urgently needed.

Cancer metabolism draws much attention from scientific community in the era of cancer immunotherapy (8–10). Many laboratory-derived parameters have been investigated as promising biomarkers in prognosis of cancer patients undergoing ICIs, such as systemic immune-inflammation index (SII) (11), prognostic nutritional index (PNI) (12), modified Glasgow Prognostic Score (mGPS) (13), C-reactive protein to albumin ratio (CAR) (14), along with neutrophil-to-lymphocyte ratio (NLR) (15). According to albumin and C-reactive protein (CRP) levels in the serum, CAR is determined to be CRP-to-albumin ratio. CAR is calculated as the following formula: CAR = CRP (mg/liter)/albumin (g/liter) (16). It is reported that the median value and normal range of CAR in healthy individuals are as follows: 0.21 (0.05–1.08) (17). Previous studies have analyzed relation of CAR with patient survival using ICIs, whereas the results were controversial (14, 18–27). For example, some researchers identified CAR to be the efficient biomarker for predicting prognosis of cancer patients receiving ICIs (18, 23–25). However, some other scholars reported that relation of CAR with prognosis among carcinoma cases using ICIs was nonsignificant (21). Therefore, we collected the most recent and comprehensive literature and carried this meta-analysis for precisely identifying CAR’s effect on predicting prognosis of carcinoma cases receiving ICIs treatment.

## Materials and methods

### Study guideline

The present meta-analysis was performed following guidelines of Preferred Reporting Items for Systematic Reviews and Meta-Analyses (PRISMA) (28).

### Literature search strategy

Through search of Web of Science, PubMed, Cochrane Library, and Embase databases was carried out. The search strategy was shown below: (“C-reactive protein to albumin ratio” or “C-reactive protein albumin ratio” or “CRP/albumin ratio” or “C-reactive protein/albumin ratio”) and (“immune check point inhibitor” or “PD-1” or “PD-L1” or “immunotherapy” or “nivolumab” or “CTLA-4” or “pembrolizumab” or “avelumab”). The search was updated on 11 December 2022. The publication language was restricted to English. Reference lists in enrolled articles were manually searched for identifying possible missing articles.

### Inclusion and exclusion criteria

Studies conforming to criteria below were included: (1) studies recruiting carcinoma patients receiving ICIs treatment, (2) CAR data before treatment were measured, (3) studies reported relation of CAR with survival outcomes among cancer cases undergoing ICIs, (4) the hazard ratios (HRs) together with 95% confidence intervals (CIs) were available or calculable by provided information, (5) identification of a threshold for stratifying high/low CAR, and (6) English articles. Studies conforming to conditions below were excluded: (1) case reports, reviews, letters, comments, conference abstracts, and editorials; (2) duplicates; (3) studies with not enough data to analyze survival; and (4) animal studies. Overall survival (OS) and progression-free survival (PFS) were treated as primary and secondary outcomes, separately.

### Data extraction and quality assessment

Two independent reviewers (M.D. and W.W.) analyzed the included articles and extracted the data. All disagreements were resolved by discussion until consensus. Data collected included first author, country, publication year, study period, sample size, age, sex, cancer type, cancer stage, treatment, ICIs antibody type, threshold CAR, follow-up, study center, HR analysis type, and HRs together with 95% CIs. Newcastle–Ottawa Scale (NOS) was adopted for assessing enrolled study methodological quality (29). Typically, NOS assesses study quality from three perspectives, including comparability (0–2 points), selection (0–4 points), and outcomes (0–3 points). Total NOS score was 0–9, while articles of NOS score ≥ 6 were defined to be high-quality ones.

### Statistical analysis

This work determined combined HRs together with 95% CIs for estimating whether CAR was efficient in predicting prognosis of OS and PFS for cancer cases receiving ICIs. Inter-study heterogeneities were assessed by Higgins I² statistics and Cochran’s Q test. *I*^2^ > 50% or *P* < 0.10 in heterogeneity indicates obvious heterogeneity, so the random-effects model was employed; or else, the fixed-effects model is used. Pooled odds ratios (ORs) and 95% CIs were calculated to estimate the impact of CAR on objective response rate (ORR). Subgroup analyses according to different factors were carried out for detecting potential heterogeneity source. We performed sensitivity analyses to examine the effects of omitting studies potentially contributing to data heterogeneity. Begg’s test and funnel plot were carried out for examining publication bias. Stata software version 12.0 (Stata Corporation, College Station, TX) was adopted for statistical analysis. *p* < 0.05 (two sided) stood for statistical significance.

### Ethnics approval

Based on already published studies, ethical approval was waived for this meta-analysis.

## Results

### Literature retrieval process

**Figure 1:**
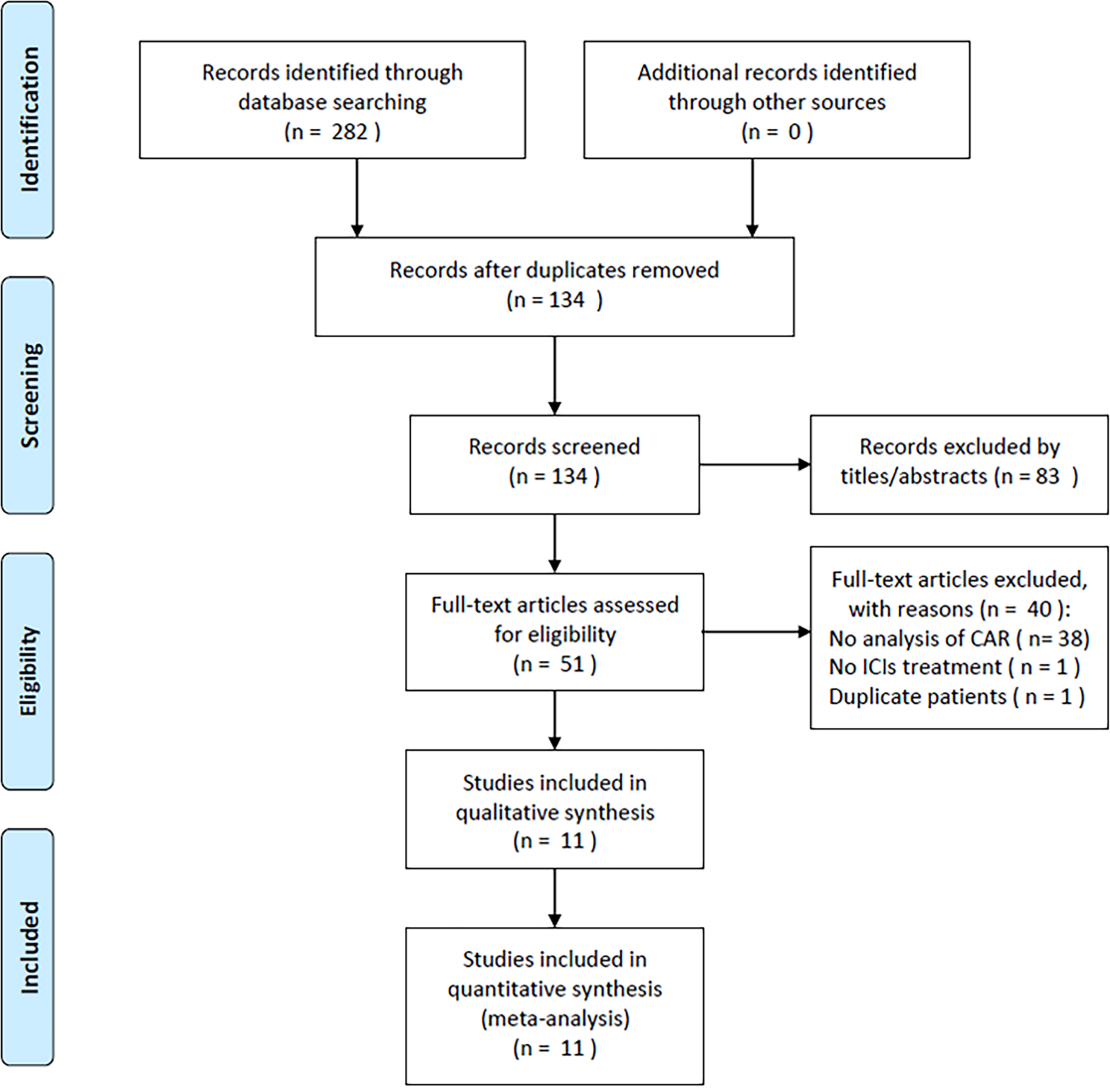
Flow diagram of the literature search and selection.

Upon primary database search, altogether 282 records were obtained, among which 134 studies were remained following removal of duplicates. Subsequently, through title and abstract screening, 83 articles were excluded, while the rest 51 were assessed through reading the full texts. Then, we discarded 40 articles due to no analysis of CAR (*n* = 38), no ICIs treatment used (*n* = 1), and duplicate patients involved (*n* = 1). Finally, the present meta-analysis included altogether 11 studies involving 1,321 cases (14, 18–27) (**Figure 1**; **Table 1**).

**Table 1:** Baseline characteristics of included studies in the meta-analysis.

Study	Year	Country	Sample size	Study period	Age (years) Median (range)	Gender (M/F)	Cancer type	Tumor stage	Treatment	ICIs type	Cutoff value	Follow-up (months) Median(range)	Survival endpoints	HR analysis	NOS score	Study center
Inoue, T.	2018	Japan	201	2015-2016	68(27-87)	135/66	NSCLC	III-IV	Nivolumab	Anti-PD-1	0.3	To Sep 2016	OS	Univariate	8	Multicenter
Kondo, T.	2019	Japan	39	2015-2017	65(28-84)	24/15	Melanoma	IV	Nivolumab	Anti-PD-1	0.057	11.9(5.0-36.1)	PFS	Univariate	6	Single center
Tamiya, M.	2019	Japan	213	2017-2018	71(39-91)	176/37	NSCLC	III-IV	Pembrolizumab	Anti-PD-1	0.3	11.0	PFS	Multivariate	8	Multicenter
Araki, T.	2021	Japan	113	2015-2019	68.5(36-86)	87/26	NSCLC	III-IV	Nivolumab	Anti-PD-1	0.83	1-48	OS, PFS	Multivariate	8	Multicenter
Ogura, Y.	2021	Japan	34	2019-2020	72(55-81)	29/5	NSCLC	III-IV	ICIs+ chemotherapy	Anti-PD-1/PD-L1	0.424	1-18	OS, PFS	Univariate	7	Single center
Takamori, S.	2021	Japan	304	2016-2019	66(31-88)	242/62	NSCLC	III-IV	Nivolumab/Pembrolizumab/ Atezolizumab	Anti-PD-1/PD-L1	0.224	1-30	OS, PFS	Univariate	8	Multicenter
Tanoue, K.	2021	Japan	46	2014-2019	66(41-87)	38/8	HNSCC	R/M	Nivolumab	Anti-PD-1	0.3	1-26	OS, PFS, ORR	Multivariate	9	Multicenter
Ikoma, T.	2022	Japan	93	2017-2021	70(38-85)	72/21	ESCC	III-IV	Nivolumab	Anti-PD-1	0.62	9.1(1-34.7)	OS, ORR	Multivariate	8	Multicenter
Inoue, H.	2022	Japan	41	2020-2022	68(51-81)	34/7	ESCC	R	Nivolumab	Anti-PD-1	0.119	9.8(1-25.7)	OS, ORR	Multivariate	7	Single center
Matsuo, M.	2022	Japan	164	2017-2020	65(23-87)	127/37	HNSCC	R/M	Nivolumab	Anti-PD-1	0.085	12.6(0.3-51.2)	OS	Multivariate	8	Single center
Yang, Z.	2022	China	73	2019-2021	57(31-75)	49/24	ICC	III-IV	Anti-PD-1	Anti-PD-1	0.3	11.2	OS	Univariate	7	Single center

NSCLC, non-small cell lung cancer; HNSCC, head and neck squamous cell carcinoma; ESCC, esophageal squamous cell carcinoma; ICC, intrahepatic cholangiocarcinoma; ICIs, immune checkpoint inhibitors; OS, overall survival; PFS, progression-free survival; HR, hazard ratio; NOS, Newcastle–Ottawa Scale; R, recurrent; M, metastatic; PD-1, programmed cell death protein-1; ORR, objective response rate.

### Enrolled study features

**Table 1** displays basic features of enrolled articles. All articles were published from 2018 to 2022, indicating the recent literature on the research topic. There were 10 articles carried out in Japan (14, 18–26), and one study was carried out in China (27). All articles were published in the English language and of retrospective design (14, 18–27), with the median sample size of 93 (range, 34-304). Five articles recruited non-small cell lung cancer (NSCLC) cases (14, 18, 20–22), two studies enrolled head and neck squamous cell carcinoma (HNSCC) cases (23, 26), two studies enrolled patients with esophageal squamous cell carcinoma (ESCC) (24, 25), while one each study included patients with melanoma (19) and intrahepatic cholangiocarcinoma (ICC) (27). Nine studies used anti-PD-1 treatment (14, 18–20, 23–27), and two studies applied anti-PD-1/PD-L1 strategy (21, 22). Thresholds of CAR were 0.057–0.83 in included studies. The median value of CAR cutoff values was 0.3, and the mean value was 0.324. Six articles were multicenter researches (14, 18, 20, 22–24), and five studies were conducted in single center (19, 21, 25–27). Nine studies reported whether CAR was important for predicting the prognosis of OS in cancer cases receiving ICIs treatment (14, 18, 21–27), and six articles investigated relation of CAR with PFS (14, 19–23). Multivariate regression from six articles (14, 20, 23–26), while univariate regression from five articles (18, 19, 21, 22, 27), reported HRs together with 95%CIs. For those enrolled articles, their NOS scores were 6–9 (median, 8), suggesting that high quality of those eligible articles.

### C-reactive protein to albumin ratio and overall survival of cancer patients receiving immune checkpoint inhibitors

**Figure 2:**
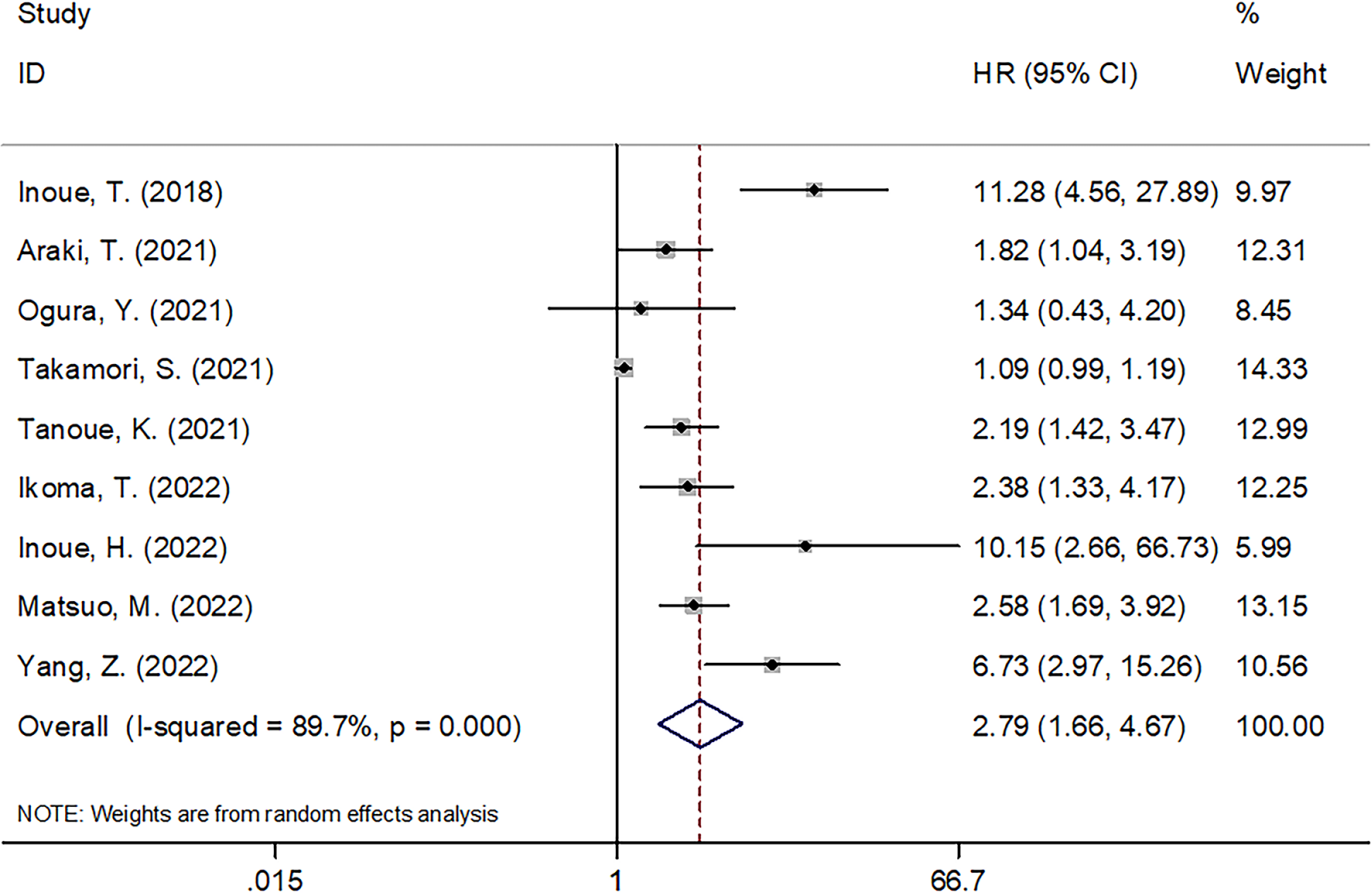
Forest plots of studies evaluating the association between CAR level and OS in cancer patients receiving ICIs.

Nine studies with 1,069 patients (14, 18, 21–27) provided the data on relation of CAR with OS among cancer cases receiving ICIs treatment. Combined HR = 2.79, 95% CI = 1.66–4.67, *p* < 0.001 were obtained, which indicated that a higher CAR level significantly predicted poor OS in carcinoma patients using ICIs (**Figure 2** and **Table 2**). This work adopted a random-effects model because of the obvious heterogeneity (*I*^2 ^= 89.7%, *Ph* < 0.001). Subgroup analysis was utilized stratified by country, sample size, cancer type, clinical stage, treatment, CAR cutoff value, HR type, and study center. As shown in **Table 2**, CAR’s role in predicting OS remained unchanged by country, sample size, clinical stage, or study center (all *p* < 0.05).

**Table 2:** Subgroup analysis of CAR and prognosis for OS in cancer patients undergoing ICIs.

Subgroups	No. of studies	No. of patients	HR (95%CI)	*p*	Effects model	Heterogeneity *I*^2^(%) *Ph*	
Total	9	1,069	2.79(1.66-4.67)	< 0.001	Random	89.7	< 0.001
Country							
Japan	8	996	2.48(1.49-4.12)	< 0.001	Random	88.6	< 0.001
China	1	73	6.73(2.97-15.26)	< 0.001	–	–	–
Sample size							
< 100	5	287	3.01(1.74-5.20)	< 0.001	Random	59.4	0.043
≥ 100	4	782	2.50(1.16-5.39)	0.019	Random	92.9	< 0.001
Cancer type							
NSCLC	4	652	2.23(0.93-5.33)	0.073	Random	89.4	< 0.001
ESCC	2	134	4.01(1.03-15.68)	0.046	Random	63.9	0.096
HNSCC	2	210	2.39(1.76-3.24)	< 0.001	Fixed	0	0.604
Others	1	73	6.73(2.97-15.26)	< 0.001	–	–	–
Clinical stage							
III-IV	6	818	2.69(1.31-5.49)	0.007	Random	90.4	< 0.001
R/M	2	210	2.39(1.76-3.24)	< 0.001	Fixed	0	0.604
IV/R	1	41	10.15(2.03-50.79)	0.005	–	–	–
Treatment							
Anti-PD-1	7	731	3.38(2.17-5.25)	< 0.001	Random	69.8	0.003
Anti-PD-1/PD-L1	2	338	1.09(1.00-1.20)	0.061	Fixed	0	0.723
Cutoff value							
< 0.3	3	509	2.26(0.94-5.43)	0.068	Random	91.1	< 0.001
≥ 0.3	6	560	3.05(1.78-5.23)	< 0.001	Random	73.3	0.002
HR analysis							
Multivariate	5	457	2.34(1.84-2.98)	< 0.001	Fixed	5.9	0.373
Univariate	4	612	3.19(0.88-11.54)	0.077	Random	93.1	< 0.001
Study center							
Single center	4	312	3.55(1.69-7.46)	0.001	Random	64.1	0.039
Multicenter	5	757	2.36(1.27-4.39)	0.007	Random	90.5	< 0.001

NSCLC, non-small cell lung cancer; HNSCC, head and neck squamous cell carcinoma; ESCC, esophageal squamous cell carcinoma; ICIs, immune checkpoint inhibitors; OS, overall survival; HR, hazard ratio; R, recurrent; M, metastatic; PD-1, programmed cell death protein-1.

### C-reactive protein to albumin ratio in progression-free survival of carcinoma patients undergoing immune checkpoint inhibitors

**Figure 3:**
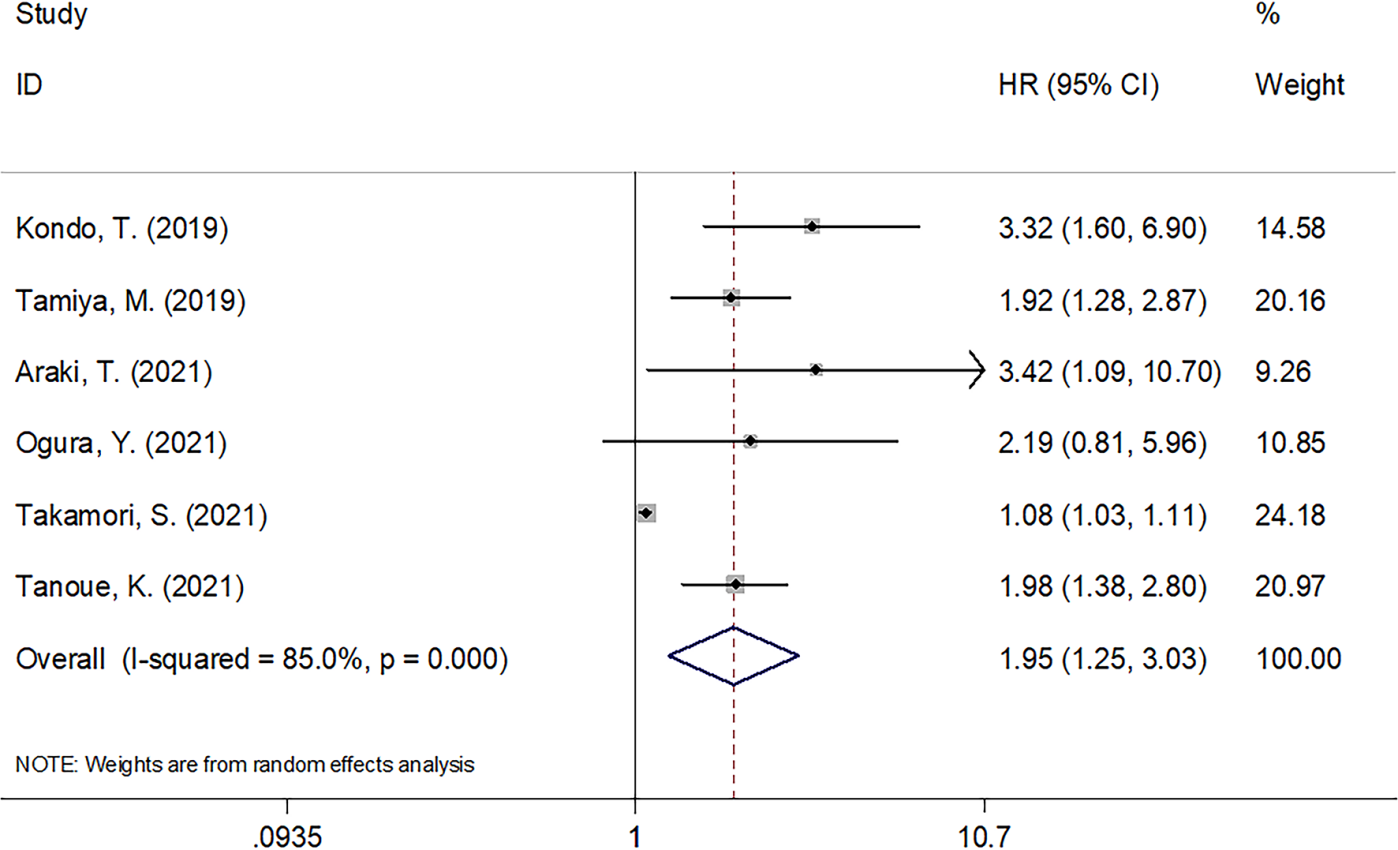
Forest plots of studies evaluating the association between CAR level and PFS in cancer patients treated with ICIs.

There were six articles involving 749 cases (14, 19–23) analyzing whether CAR was significant in predicting PFS among cancer patients receiving ICIs. According to **Table 3** and **Figure 3**, combined results included HR = 1.95, 95% CI = 1.25–3.03, *p* = 0.003, indicating a significant relation of CAR with worse PFS among carcinoma cases receiving ICIs treatment. According to subgroup analysis, CAR’s role in predicting PFS remained significant irrespective of cancer type, clinical stage, ICIs treatment, or study center (**Table 3**).

**Table 3:** Subgroup analysis of CAR and prognosis for PFS in cancer patients undergoing ICIs.

Subgroups	No. of studies	No. of patients	HR (95% CI)	*p*	Effects model	Heterogeneity *I*^2^(%) *Ph*	
Total	6	749	1.95(1.25-3.03)	0.003	Random	85.0	< 0.001
Sample size							
< 100	3	119	2.18(1.61-2.96)	< 0.001	Fixed	0	0.459
≥ 100	3	630	1.62(0.91-2.86)	0.099	Random	82.8	0.003
Cancer type							
NSCLC	4	664	1.69(1.01-2.82)	0.045	Random	77.8	0.004
HNSCC	1	46	1.98(1.39-2.82)	< 0.001	–	–	–
Others	1	39	3.32(1.60-6.89)	0.001	–	–	–
Clinical stage							
III-IV	4	664	1.69(1.01-2.82)	0.045	Random	77.8	0.004
R/M	1	46	1.98(1.39-2.82)	< 0.001	–	–	–
IV/R	1	39	3.32(1.60-6.89)	0.001	–	–	–
Treatment							
Anti-PD-1	4	411	2.13(1.67-2.72)	< 0.001	Fixed	0	0.476
Anti-PD-1/PD-L1	2	338	1.08(1.04-1.12)	< 0.001	Fixed	48.1	0.165
Cutoff value							
< 0.3	2	343	1.78(0.60-5.32)	0.301	Random	88.9	0.003
≥ 0.3	4	406	2.02(1.57-2.60)	< 0.001	Fixed	0	0.822
HR analysis							
Multivariate	3	372	2.01(1.55-2.61)	< 0.001	Fixed	0	0.641
Univariate	3	377	1.85(0.82-4.18)	0.138	Random	81.7	0.004
Study center							
Single center	2	73	2.87(1.59-5.18)	< 0.001	Fixed	0	0.510
Multicenter	4	676	1.71(1.06-2.74)	0.027	Random	86.7	< 0.001

NSCLC, non-small cell lung cancer; HNSCC, head and neck squamous cell carcinoma; ESCC, esophageal squamous cell carcinoma; ICIs, immune checkpoint inhibitors; PFS, progression-free survival; HR, hazard ratio; R, recurrent; M, metastatic; PD-1, programmed cell death protein-1.

### Association between C-reactive protein to albumin ratio and objective response rate

**Figure 4:**
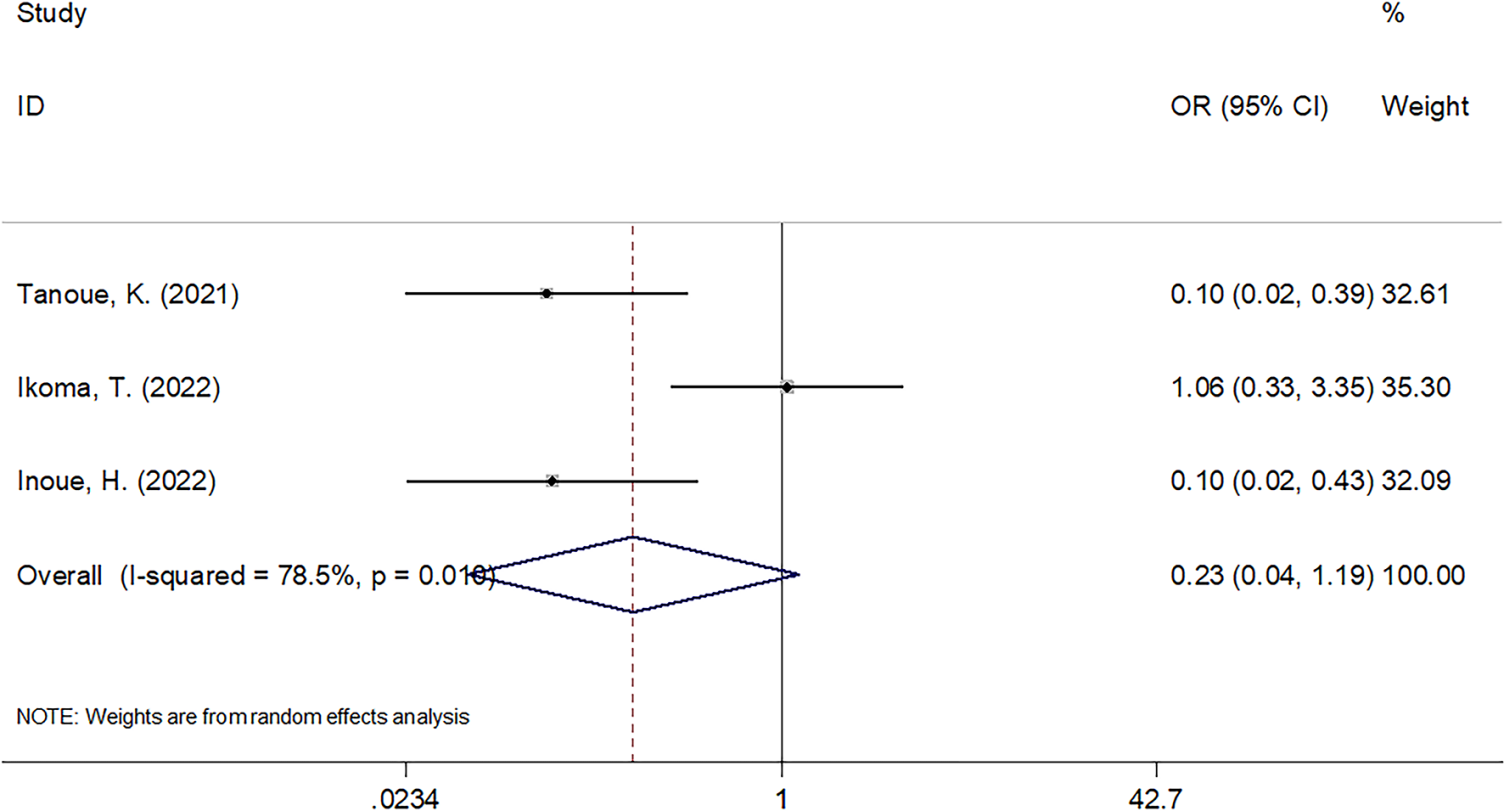
The forest plots of studies evaluating the association between CAR level and ORR in cancer patients treated with ICIs.

A total of three studies with 180 cases (23–25) reported the impact of CAR on ORR in cancer patients undergoing ICIs. Due to significant heterogeneity (*I*^2 ^= 78.5%, *Ph* = 0.010), a random-effects model was used. The pooled results were as follows: OR = 0.23, 95% CI = 0.04–1.19, *p* = 0.079, suggesting that there was no significant association between CAR and ORR (**Figure 4**).

### Sensitivity analysis

**Figure 5:**
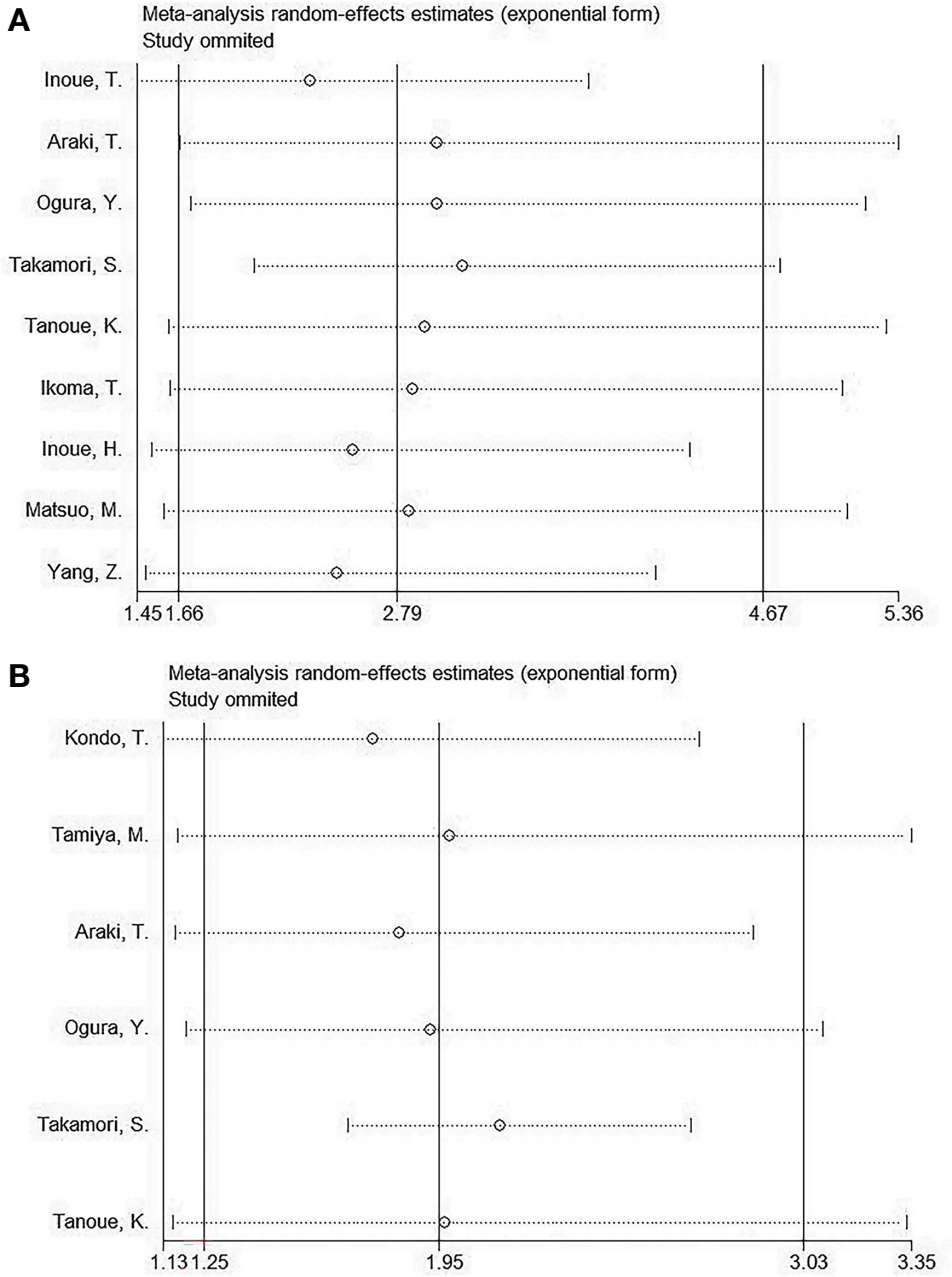
Sensitivity analysis. **(A)** OS; **(B)** PFS.

This work conducted sensitivity analysis through omitting a work each time for testing our result robustness. According to **Figure 5**, OS and PFS HR estimates did not significantly change, suggesting that our meta-analysis was credible.

### Publication bias

**Figure 6:**
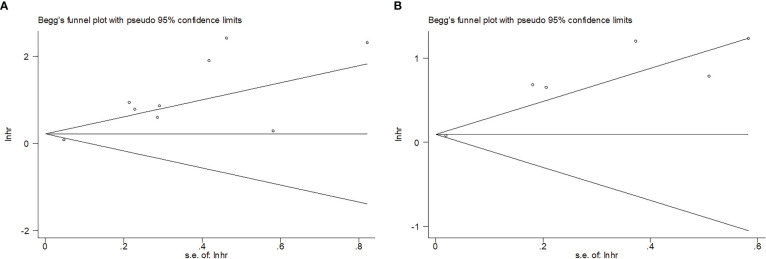
Begg’s funnel plot of publication bias test. **(A)** OS, *p* = 0.466; **(B)** PFS, *p* = 0.851.

Funnel plots and Begg’s test were performed to test possible publication bias. According to **Figure 6**, the funnel plots were symmetrical and Begg’s test revealed no evidence of obvious publication bias for OS (*p* = 0.466) or PFS (*p* = 0.851).

## Discussion

CAR’s effect on prognosis prediction for cancer cases receiving ICIs treatment remains controversial in prior works. According to this current meta-analysis, data from 11 articles involving 1,321 cases were synthesized (14, 18–27) to accurately identify the association of CAR with cancer cases receiving ICIs. According to this meta-analysis, the high CAR level markedly predicted the poor OS and PFS among ICI-treated carcinoma cases. Moreover, the prognostic effect of CAR was not influenced by clinical stage and study center for OS or PFS. Publication bias test and sensitivity analysis suggested that our results were reliable. Collectively, in the present meta-analysis, elevated CAR was a reliable and cost-effective marker for poor survival in cancer patients undergoing ICIs. Monitoring CAR level could aid in identifying high-risk ICIs treated patients and therefore help tailor treatment strategy. As far as we know, the present meta-analysis was the first to explore CAR for its value in prognosis prediction for cancer cases receiving ICIs.

CAR was computed by CRP to albumin ratio; consequently, an increased CAR is attributed to the increased CRP level and/or the decreased serum albumin level. The mechanisms of CAR for prognosis of ICI-treated cancer patients are not comprehensively analyzed hitherto; however, it is interpreted as follows. First, CRP is synthesized in the liver and plays a role in acute inflammation (30). CRP is a pro-inflammatory protein regulated *via* numerous pro-inflammatory factors, containing IL-1 and IL-6 (31). As a result of elevated serum CRP levels, vascular endothelial growth factor levels often increase as well, contributing to the progression and formation of cancers (32). Second, serum albumin accounts for approximately 60% of the total protein; therefore, albumin level can be usually recognized to be the biological indicator of nutritional status (33). In many cancers, there is a significant correlation between serum albumin levels and systemic inflammatory response, body nutritional status, as well as clinical survival (34). As a result of cancer-related inflammation or infection, serum albumin escapes into the interstitium through increased capillary permeability, which also leads to hypoalbuminemia (35). Therefore, the CAR combines serum CRP and albumin levels concurrently, can more accurately reflect the host’s inflammatory status, and can participate in prediction of survival outcomes in ICI-treated cancer patients. Notably, CAR is derived from blood test. These parameters are easily available and cost effective. No additional cost was added for patients, because blood test is a routine test in clinics. Therefore, CAR is cost effective for patients and clinicians.

Many recent articles report that CAR can be used to predict prognosis of different cancers based on meta-analysis (36–39). Wu et al. showed that the large CAR before treatment effectively predicted dismal outcome of urinary cancer cases based on a meta-analysis including 2,941 cases (36). Our previous work reported that the high CAR showed marked relation with dismal OS together with decreased disease-free survival (DFS) or recurrence-free survival among bile duct cancer cases through the meta-analysis comprising fourteen articles (38). According to one meta-analysis involving 771 cases, the large CAR negatively predicted the prognosis of metastatic colorectal cancer cases (40). Luan et al. demonstrated that patients with head and neck cancer have a poorer prognosis when their pretreatment CAR is elevated by a meta-analysis containing 7,080 participants (41). Xie et al. carried out the meta-analysis involving 2,271 subjects, according to their results, pancreatic cancer patients with elevated CAR had inferior OS, PFS, and DFS (42). In this meta-analysis, high CAR was recognized to be the significant prognostic biomarker for carcinoma patients treated with ICIs, which was in line with findings of previous studies on diverse cancers.

Certain limitations should be pointed out in the present meta-analysis. Initially, all enrolled articles are from east Asia, especially Japan (10/11). There was no restriction on geographical region of enrolled articles. Second, cutoff values of CAR were not consistent among eligible studies. The cutoff values are various across included studies from 0.057 to 0.83. The cutoff values can be influenced by the recruiting subjects and the determination methods. We adopted the CAR = 0.3 for subgroup analysis, because the median value of CAR cutoff was 0.3 of included studies. Third, the enrolled articles were of retrospective nature, possibly leading to inherent selection bias. Fourth, the units of CAR have not been standardized across studies. Some studies used the formula: CAR = CRP (mg/liter)/albumin (g/liter) (16). Whereas some other used CAR = CRP (mg/dl)/albumin (g/dl) (43). Although the CAR ratio was not influenced by the units: (mg/liter)/(g/liter) or (mg/dl)/(g/dl). Due to these limitations, large, prospective clinical studies are still warranted for validating our results.

In summary, the high CAR level before treatment significantly predicted inferior OS and PFS among cancer cases undergoing ICIs. The prognostic effect of CAR was not influenced by clinical stage or study center for ICI-treated cancer cases. CAR is easily available and cost effective and serves as the potential biomarker for selecting cancer cases benefiting from ICIs.

## Data availability statement

The original contributions presented in the study are included in the article/supplementary material. Further inquiries can be directed to the corresponding author.

## Author contributions

MD and WW conceived the study concept and design. MD and WW were responsible for statistical analysis. MD and WW helped the data analysis and consultation for manuscript preparation. MD wrote the first draft. WW revised the manuscript. All authors made the critical revision of the manuscript for important intellectual content. All authors contributed to the article and approved the submitted version.
